# 3D facial data analysis and replaceable mold design for customizing CPAP nasal mask cushions

**DOI:** 10.1016/j.bjorl.2026.101852

**Published:** 2026-06-18

**Authors:** Ding Yang Hsu

**Affiliations:** Ming-Chi University of Technology, Department of Industrial Design, New Taipei City, Taiwan

**Keywords:** Obstructive sleep apnea, CPAP therapy, 3D facial data analysis, Customized nasal mask, Rapid mold design

## Abstract

**Objective(s):**

This study aimed to develop and evaluate a novel method for customizing CPAP nasal mask cushions to improve patient comfort, mask fit, and adherence to therapy for individuals with Obstructive Sleep Apnea (OSA).

**Methods:**

A customized CPAP nasal mask cushion design was developed using a combination of 3D facial data analysis and a replaceable rapid mold system. Twenty moderate OSA patients were enrolled for facial scanning and design analysis, and five representative participants were selected for validation testing using a within-subject comparison between their original factory-fitted cushions and the customized versions. Principal Component Analysis (PCA) to identify key measurement points influencing mask design. A pocket-style mold system with interchangeable cores enabled the efficient fabrication of patient-specific cushions. Objective assessments and patient-reported outcomes were used to compare the customized cushions with conventional designs in terms of comfort, strap tension, facial pressure, and air leakage.

**Results:**

Customized nasal mask cushions demonstrated significant improvements over factory-fitted designs. Patient-reported comfort scores increased from 6.2 ± 0.8 to 8.8 ± 0.6 (p < 0.01), while scores for strap tension and facial pressure improved from 6.5 ± 0.9 to 9.0 ± 0.7 (p < 0.01). Patients also reported a substantial reduction in air leakage and a preference for the customized designs for long-term use.

**Conclusion:**

The findings indicate that customized CPAP nasal mask cushions effectively enhance fit and comfort, addressing key barriers to CPAP adherence. This method provides a scalable and cost-effective solution leveraging 3D scanning and rapid mold technologies. Further research involving larger sample sizes, longer durations, and objective performance metrics will be necessary to assess clinical applicability and long-term adherence outcomes

## Highlights

• 3D facial data analysis improves CPAP nasal mask fit and comfort for OSA patients.
• Replaceable rapid mold design enables efficient production of customized cushions.
• Customized cushions reduce facial pressure, strap tension, and air leakage.
• Patients prefer personalized CPAP cushions for long-term use and adherence.

## Introduction

Sleep is a fundamental requirement for restoring bodily functions and is crucial in emotional regulation and cognitive memory restructuring. However, sleep disorders, particularly Obstructive Sleep Apnea (OSA), are highly prevalent globally. Research indicates that approximately one-third of Western adults and 15% of Taiwanese adults suffer from OSA, with many experiencing snoring. Untreated OSA can lead to severe health complications, including hypertension, heart failure, and sudden death.

CPAP therapy is the most effective treatment for OSA, delivering continuous air pressure through a nasal mask to open obstructed airways. However, the acceptance of CPAP masks among patients is limited due to issues such as inadequate seal, discomfort, and inconvenience, causing many to discontinue treatment. Recent studies have shown that non-adherence rates to CPAP therapy range between 29% and 50%, with approximately 70% of patients citing mask-related issues ‒ such as discomfort, poor fit, or air leakage ‒ as the primary reasons for discontinuation.^1^^,^^2^ This pilot study aims to address the limitations of current mask designs by proposing an innovative method for customizing nasal mask cushions, improving the patient user experience.

OSA significantly impacts patients' health and quality of life. Research shows that untreated OSA can result in numerous health risks, including cardiovascular diseases and sudden death. While CPAP therapy remains the gold standard for OSA treatment, patient adherence is often low.

Collard et al.^3^ surveyed over 20 OSA patients using CPAP for over a year, revealing that average daily usage ranged from 5 to 6.5 h. Approximately 25% of patients discontinued therapy, citing reasons such as nasal congestion (34%), inconvenience (28%), and mask leakage or discomfort (20%). Rabec et al. (2004) further emphasized that mask leakage is one of the most common problems in CPAP therapy, leading to frequent awakenings and reduced treatment efficacy.

**Fig. 1:**
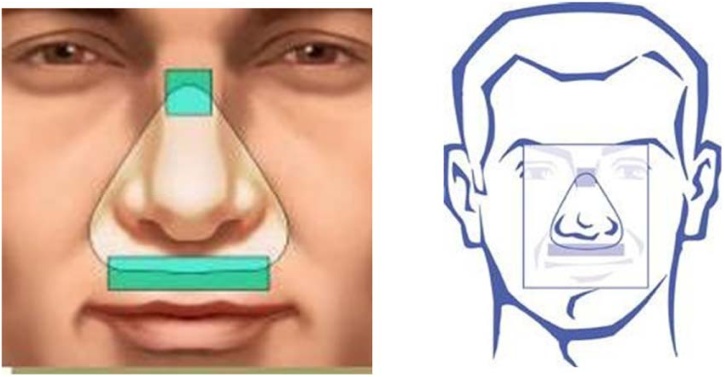
Triangular points for nasal mask fitting.

Current commercial nasal mask cushions are typically available in standardized sizes (e.g., S, M, L), providing limited options that fail to accommodate diverse facial geometries. Cheng et al.^4^ demonstrated that customized nasal mask designs significantly improved patient adherence to CPAP therapy, especially during long-term treatment (Fig. 1).

Hsu et al.^5^ proposed using 3D scanning and 3D printing technology to manufacture customized nasal mask cushions, greatly enhancing design precision and comfort. However, this approach faces challenges in mass production and cost efficiency. Building on this foundation, the present study introduces an innovative method utilizing replaceable rapid molds and 3D facial data analysis to address these limitations, enabling the rapid production of personalized nasal mask cushions.

The IEK report from Taiwan's Industrial Technology Research Institute highlights that the future of medical devices lies in miniaturization, portability, wireless technology, and customization.^7^ The widespread adoption of 3D printing supports customized design, particularly in resolving issues of inadequate seal and discomfort. Developing personalized CPAP nasal mask cushions for OSA patients meets clinical needs and aligns with industry trends.

## Methods

### Participant selection and facial data collection

**Fig. 2:**
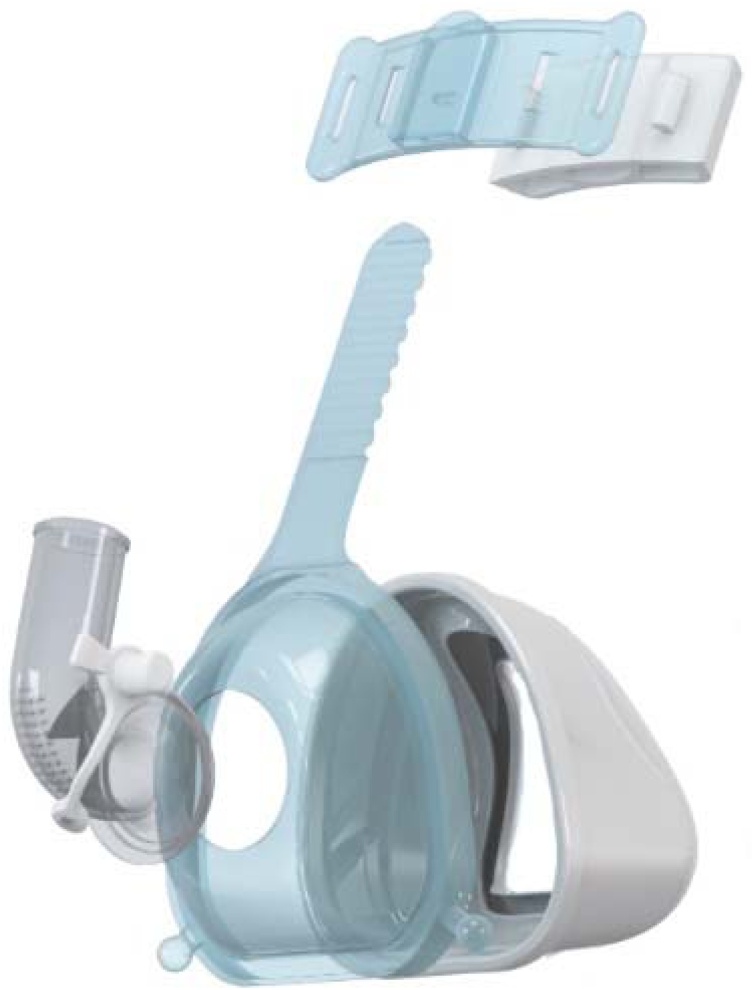
DeZire™ Lite Nasal Masks.

Twenty participants diagnosed with moderate Obstructive Sleep Apnea (OSA) were recruited for 3D facial scanning and nasal mask cushion design. The inclusion criteria were: (1) Aged 30–65 years; (2) Currently using a DeZire™ Lite Nasal Mask (Fig. 2, Galemed CO., Taiwan, CPAP nasal mask with a replaceable cushion), size S; (3) No history of facial trauma, burns, deformities, or surgery.

Exclusion criteria included: (1) Craniofacial abnormalities; (2) Severe respiratory or neurological conditions; and (3) Previous intolerance to CPAP therapy.

Five of the 20 participants were selected to proceed to the experimental validation phase.

**Fig. 3:**
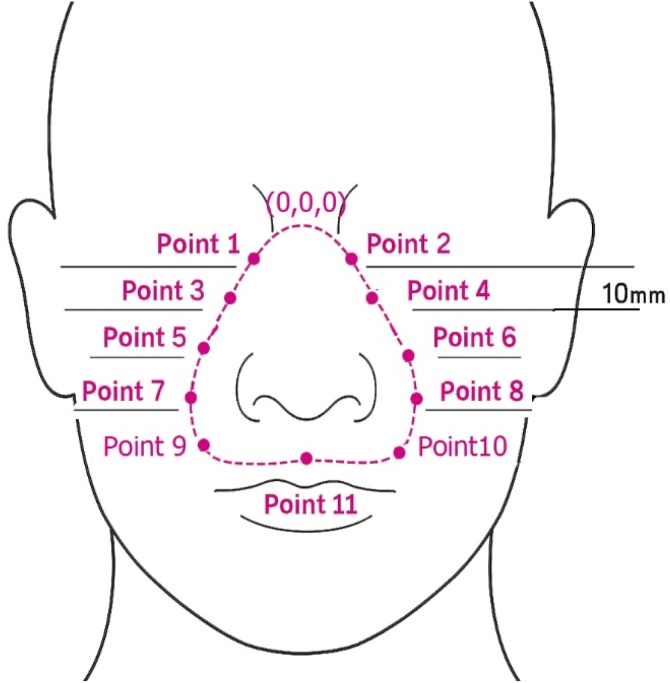
11-landmarks.

The study set the nasal root as the origin (x, y, *z* = 0.0.0) and defined three axes of 11 landmarks at 10 mm intervals along the mask shape (Fig. 3).

**Fig. 4:**
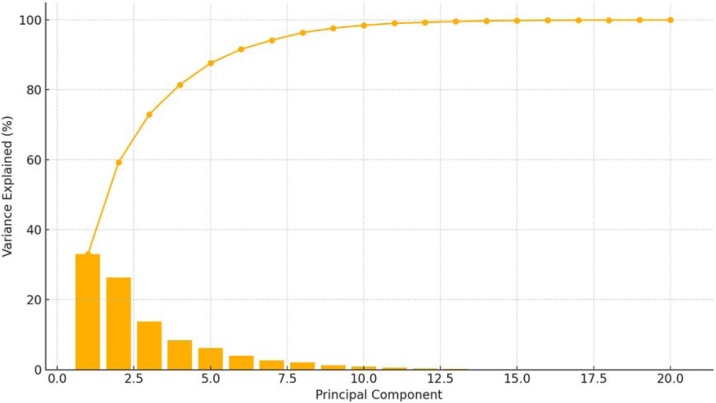
Scree plot and cumulative variance.

Landmark Analysis and Participant Selection for Customization. Principal Component Analysis (PCA) was performed on the 20 × 33 standardized coordinate matrix (11-landmarks × three axes). The resulting eigenvalue spectrum is shown in Fig. 4. The first three principal components explained 33.2%, 24.8%, and 15.1% of the total variance for a cumulative 73.1% (Fig. 4).

**Fig. 5:**
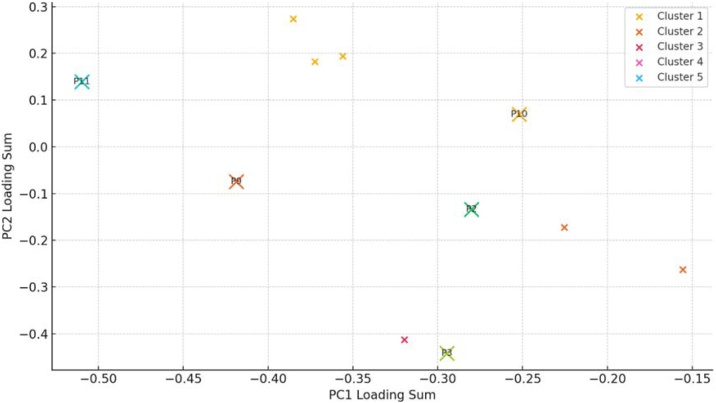
Point loading in PC1-PC2 space clusters.

Variance bars explained by each principal component (bar) and cumulative variance (line) plane. We plotted each point’s loading vector in the PC1–PC2 plane to identify the most informative landmarks. We plotted each point’s loading vector in the PC1–PC2 plane to identify the most informative landmarks and applied K-means clustering (K = 5) (Fig. 5). Points 1 and 2 clustered together, reflecting nasal-bridge geometry (PC1), while Points 9 and 10 formed a second cluster associated with nasal depth and philtrum position (PC2). Point 11 emerged as an independent cluster corresponding to lateral facial contour (PC3).

Loading vectors of all 11 landmarks in the PC1–PC2 space. Colors denote K-means clusters; bold markers indicate the five representative core points (1, 2, 9, 10, 11). When re-running PCA using only these five core points, they collectively accounted for 72.98% of the total variance, justifying the omission of Points 3–8 from subsequent measurements.

### Customized cushion design and mold fabrication

**Fig. 6:**
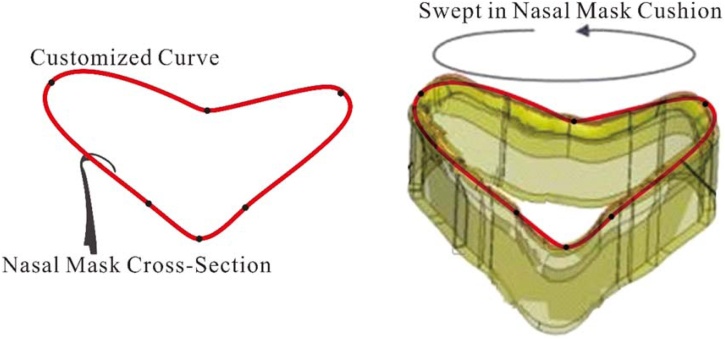
(A) Nasal mask cross-section and customized curve, (B) Swept design.

Using parametric modeling techniques, the customized curves were connected to design a cushion surface closely conforming to the patient’s facial contours. The nasal mask cross-section was combined with the customized curve, and the entire cushion design was created along this scanned curve, as shown in Fig. 6A. The cushion is the primary component in contact with the human body, making it critical for overall comfort.

### Rapid mold design and production

**Fig. 7:**
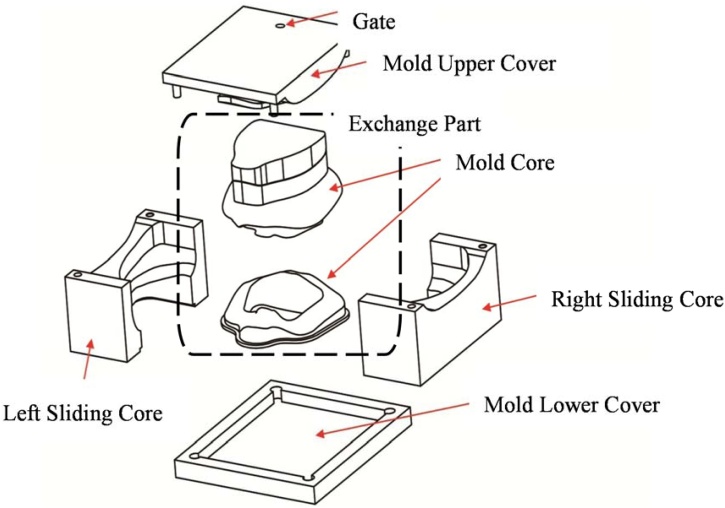
Pocket-style rapid mold design.

**Fig. 8:**
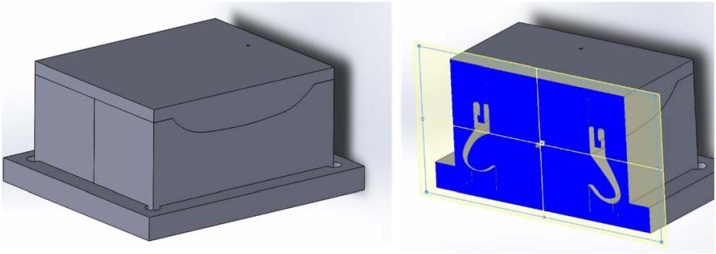
(A) Assembly mold, (B) Section view.

This study employed a pocket-style rapid mold design concept to reduce production costs and time, as illustrated in Fig. 7. The rapid mold of an upper and lower cover, right and left sliding cores, and two interchangeable mold cores. These interchangeable cores accommodate different patient-specific designs, enabling the production of customized cushions for various individuals simply by replacing the cores. Fig. 8 shows the fully assembled mold and a sectional view, highlighting the internal cavity and how the interchangeable mold cores define the cushion shape.

**Fig. 9:**
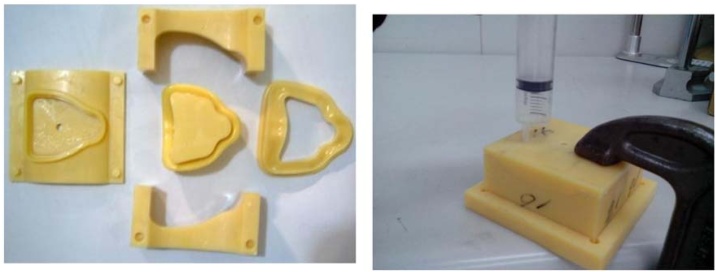
Silicone injection process.

**Fig. 10:**
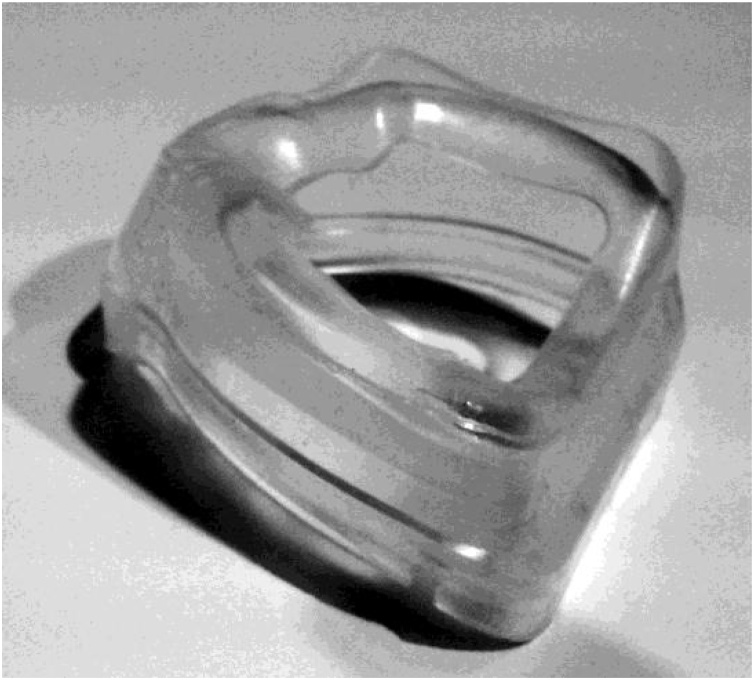
Customized cushion.

The molds were made from Chimei ABS-707, and the silicone cushions were manufactured using biocompatible silicone materials. CNC machining technology was used to create the molds, and the silicone was injected into the mold to form the cushions, as shown in Fig. 9. Fig. 10 presents the final customized nasal mask cushion after demolding, demonstrating the personalized geometry derived from each patient’s facial scan.

Experimental Validation Design. The five selected participants were tested using a within-subjects design. The five selected participants were tested using a within-subjects design. Each subject wore their original factory-fitted cushion (control condition) for 10 min, followed by the customized cushion (intervention condition) for another 10 min. Participants were unaware of the cushion type during testing. Subjective ratings and preference surveys were collected after each session.

### Evaluation metrics

1 Comfort Assessment: A 10-point scale was used, where one indicated “very uncomfortable” and 10 showed “very comfortable”. Each participant provided scores after 10-minutes of use.
2 Strap Tension and Facial Pressure Ratings: A 10-point scale was also used, with one indicating “very uncomfortable” and 10 indicating “very comfortable”. Ratings were collected after each session.
3 Patient Acceptance and Preference Survey: Participants answered three questions:
• Which mask felt more comfortable to wear?
• Which mask provided a better fit?
• Which mask would you prefer for long-term use?

## Results

This study demonstrated that customized CPAP nasal mask cushions significantly improved comfort, strap tension, and facial pressure distribution compared to factory-fitted designs.

In the comfort evaluation, factory-fitted nasal mask cushions scored an average of 6.2 ± 0.8, while the customized cushions improved this score to 8.8 ± 0.6. Participants noted that the customized cushions reduced pressure on the nasal bridge and wings, providing a softer and more comfortable fit.

In the strap tension and facial pressure evaluation, the customized cushions scored an average of 9.0 ± 0.7, compared to 6.5 ± 0.9 for factory-fitted cushions. Participants reported that factory-fitted masks required tighter straps to ensure a proper seal, resulting in higher localized pressure on the nasal bridge and wings. Customized cushions, designed to match individual facial geometries, distribute pressure more evenly and reduce discomfort in high-pressure areas.

In the acceptance and preference survey, all participants stated that the customized cushions were more comfortable and better fitting. All participants also preferred the customized cushions for long-term use, citing improved fit and reduced air leakage as key advantages.

## Discussion

The results of this study highlight the effectiveness of customized nasal mask cushions in addressing the limitations of factory-fitted CPAP mask designs. By utilizing 3D facial data analysis and Principal Component Analysis (PCA), this study identified critical measurement points, such as the nasal bridge and wings, that significantly impact mask design. Customized cushions were tailored to the facial geometries of participants, leading to a better fit and reduced pressure concentration.

The reduced strap tension required to achieve an effective seal was a significant improvement. Factory-fitted masks often rely on higher strap tension, creating discomfort and increasing the risk of skin irritation or pressure sores, particularly in the nasal bridge and wing areas. Customized cushions provide a better fit and ensure an effective seal with reduced tension, improving overall comfort and usability.

This study also introduced a replaceable rapid mold system for efficiently producing customized cushions. This system offers a cost-effective and scalable solution by utilizing interchangeable cores, allowing manufacturers to make personalized designs without extensive tooling or production delays.

The findings are consistent with prior studies. For example, Rabec et al. (2004) identified mask leakage and discomfort as key barriers to CPAP adherence. Collard et al.^6^ emphasized improving comfort to enhance long-term adherence. Cheng et al.^4^ and Hsu et al.^5^ previously demonstrated the feasibility and benefits of 3D scanning and customized designs. The present study builds on these insights by integrating rapid mold technology, further addressing production challenges and cost concerns.

Although promising, this study has limitations. The small sample size (n = 5) limits the generalizability of the results, and the short evaluation period (10-minutes per session) may not fully capture long-term usability and performance. Future research should expand the sample size and duration to validate these findings.

## Conclusion

As a pilot feasibility study, this research successfully developed and evaluated a novel method for producing customized CPAP nasal mask cushions by combining 3D facial data analysis with a replaceable rapid mold system. The results demonstrated that the customized cushions significantly outperformed factory-fitted counterparts in several key areas, including wearer comfort, strap tension, facial pressure distribution, and perceived air leakage. Participants consistently reported that the customized cushions offered a better fit and were strongly preferred for long-term use due to reduced discomfort and improved sealing performance.

The proposed method offers a scalable, cost-effective solution to two of the most common barriers to CPAP adherence ‒ mask discomfort and air leakage. By leveraging advanced 3D scanning and rapid mold manufacturing technologies, the system aligns well with contemporary trends in medical device development, particularly those focused on patient-specific customization, production efficiency, and precision medicine.

While the present study included only five participants and used a short testing duration (10-minutes per condition), the findings provide valuable foundational evidence supporting the feasibility and clinical potential of a personalized nasal cushion system. We acknowledge the limitations of this pilot study, including the small sample size, the short duration of use, and the exclusive reliance on subjective outcome measures (such as comfort ratings and user preferences), without incorporating objective physiological data.

Future studies will build upon this framework by recruiting a larger and more diverse participant cohort, expanding the duration of CPAP use in testing (e.g., overnight or multi-day trials), and integrating objective outcome measures. These may include quantitative air leakage data recorded directly from the CPAP device, Apnea-Hypopnea Index (AHI) monitoring, and Polysomnography (PSG) assessments conducted in sleep laboratories. Further exploration of different materials for cushion fabrication may also offer additional benefits in terms of comfort, durability, and biocompatibility.

In conclusion, this study establishes a viable design and manufacturing workflow for customized CPAP nasal mask cushions, demonstrating substantial improvements in patient-perceived outcomes. The proposed system provides not only a promising direction for enhancing user comfort and CPAP adherence but also a practical roadmap for advancing the clinical translation and commercialization of personalized respiratory interfaces.

## Funding

This research did not receive any specific grant from funding agencies in the public, commercial, and not-for-profit sectors.

## Data availability statement

The authors declare that all data are available in repository.

## Declaration of competing interest

The authors declare no conflicts of interest.
